# Spectrophotometric and Chromatographic Simultaneous Estimation of Amitriptyline Hydrochloride and Chlordiazepoxide in Tablet Dosage Forms

**DOI:** 10.4103/0250-474X.57305

**Published:** 2009

**Authors:** Sejal Patel, N. J. Patel

**Affiliations:** S. K. Patel College of Pharmaceutical Education and Research, Department of Pharmaceutical Chemistry, Ganpat University, Kherva, Mehsana-382711, Gujarat, India

**Keywords:** Amitriptyline HCl, chlordiazepoxide, first derivative spectrophotometry, RP-HPLC, HPTLC, densitometric measurement

## Abstract

A binary mixture of amitriptyline HCl and chlordiazepoxide was determined by three different methods. The first method involved determination of amitriptyline HCl and chlordiazepoxide using the first derivative spectrophotometric technique at 219 and 230 nm over the concentration ranges of 1-20 and 2-24 μg/ml with mean accuracies 100.9±0.87 and 99.2±1.0%, respectively. The second method was reversed-phase high performance liquid chromatography using methanol: acetonitrile: 0.065 M ammonium acetate buffer (50:20:30, v/v/v), final pH adjust to 5.5 ± 0.02 with ortho phosphoric acid as the mobile phase and was pumped at a flow rate of 1.0 ml/min. Quantification was achieved with ultraviolet detection at 240 nm over concentration ranges of 0.25-4 and 0.1-1.6 μg/ml; mean accuracies were 100.55±0.62 and 100.71±0.81%, respectively. The third method utilized high performance thin layer chromatography method in tablet dosage form. The method was based on separation of the two drugs followed by densitometric measurements of their spots at 240 nm. The separation was carried out on Merck thin layer chromatographic aluminium sheets of silica gel 60 F254 using carbon tetrachloride: acetone: triethylamine (6:3:0.2, v/v/v) as mobile phase. The linearity was found to be in the range of 50-600 and 20-240 ng/spot for amitriptyline hydrochloride and chlordiazepoxide, respectively. The methods were successively applied to pharmaceutical formulation because no chromatographic interferences from the tablet excipients were found. The suitability of these methods for the quantitative determination of the compounds was proved by validation.

Amitriptyline HCl (AMI) is chemically, 3-(10,11-Dihydro-5H-dibenzo [a, d] cyclohepten-5-ylidene)-N,N-dimethyl-1-propanamine[1]. It is a tricyclic antidepressant used in case of anxiety and also exerts an anticholinergic activity[2]. Chlordiazepoxide (CLR) is chemically, 7-chloro-N-methyl-5-phenyl-3H-1,4-benzodiazepin-2-amine 4-oxide[1]. It is an anxiolytic agent and also a poor anticonvulsant[2]. AMI is official in IP, BP and USP. The IP[3], BP[4] and USP[5] describe HPLC, non-aqueous titration and titrimetric methods, respectively for estimation of AMI. A literature survey revealed comparison of HPLC and fluorescence polarization immunoassay method[6], determination by UV spectrophotometric method[7], dissolution studies[8] of AMI with other antipsychotic agents like nortriptyline, imipramine. HPLC determination with its major metabolites in human blood was also reported[9]. CLR is official in IP, BP and USP. The IP[3], BP[4] and USP[5] describe non-aqueous titration, potentiometry titration and HPLC methods, respectively for estimation of chlordiazepoxide. Literature survey revealed determination of major impurity of CLR by UV method[10] and HPLC method with FTIR and UV detection[11] in formulations. Spectrophotometric[12], difference spectrophotometric[13], micellar liquid chromatography[14], derivative spectrophotometry[15] methods were also reported for CLR with other drugs in pharmaceutical formulations. AMI and CLR are formulated together in the form of a tablet. Literature survey revealed simultaneous equation method for simultaneous determination of the two drugs[16]. The purpose of this study was to determine both drugs concurrently by simple, accurate, rapid and precise first derivative spectrophotometric, RP-HPLC and HPTLC assays for routine analysis.

All absorption spectra and derivatives were recorded with a UV-1700 PC UV/Vis double beam spectrophotometer with spectral width of 2 nm, wavelength accuracy of 0.5 nm and a pair of 10 mm matched quartz cells (Shimadzu, Japan). RP-HPLC chromatography was performed on a Shimadzu (Columbia, MD) RP-HPLC instrument (LC-10AT vp) equipped with UV/Vis detector, manual injector with 20-μl volume injection loop. A Phenomenex (Torrance, CA) C18 column (250×4.6 mm id, 5 μm particle size) was used as stationary phase. For HPTLC, a Camag system comprising of Linnomat V automatic sample applicator, Camag microlitre syringe, Camag TLC Scanner-3, Camag Win CAT software with stationary phase precoated silica gel 60F_254_ were used. CP224S analytical balance (Sartorius) and ultra sonic cleaner (Frontline FS 4) were used throughout the practical. Pure samples of AMI and CLR were kindly supplied by Torrent Pharmaceuticals Ltd, Ahmedabad, India. Marketed tablets of Triline Plus (Tripada Healthcare, Batch No. MT 1037) each tablet containing 25 mg AMI and 10 mg CLR were used. Triple distilled water, methanol, acetonitrile (HPLC grade, S. D. Fine Chemical, Ahmedabad, India), ammonium acetate, ortho phosphoric acid, carbon tetrachloride, acetone, triethyl amine, methanol (AR grade, S. D. Fine Chemical, Ahmedabad, India) were used.

For the first derivative spectrophotometry method, AMI and CLR stock solutions (0.5 mg/ml, each) were prepared in methanol. These stock solutions (2 ml) were transferred in to 2 separate 10 ml measuring flasks and diluted to the mark with methanol to a final concentration of 100 μg/ml each. Considering all the derivative order spectra of AMI and CLR from first to fourth derivative, the first derivative order spectra with d(N)= 2 was found suitable. The zero crossing point on the first derivative spectra of one drug, the other drug shows substantial absorbance, these two wavelengths can be employed for the estimation of AMI and CLR without any interference from other drug in combined formulations. From the derivatised spectra of prepared mixtures the absorbances were measured at 219 nm for AMI and 230 nm for CLR. These absorbances Vs concentration were plotted in the quantitative mode to obtain the working curves from which by extrapolating the value of absorbances of the sample solution, the concentration of the corresponding drugs were determined. Both the drugs obeyed Beer's Law.

For the RP-HPLC method, AMI and CLR stock solutions (100 μg/ml and 40 μg/ml, respectively) were prepared in methanol. These solutions (0.5 ml) were transferred into 2 separate 10 ml volumetric flasks and diluted to the mark with methanol to give final concentrations of 5 and 2 μg/ml, respectively. Accurate aliquots of 0.25, 0.5, 1.0, 2.0 and 4.0 ml AMI and CLR, from their working solutions (5 and 2 μg/ml) were transferred into a separate 5 ml volumetric flasks, diluted to volume with methanol. Using the Shimadzu instrument, chromatogram was recorded using a flow rate, 1 ml/min at ambient temperature and the eluent monitored at 240 nm. The separation was done on a C18 column using methanol:acetonitrile:0.065 M ammonium acetate buffer (50:20:30, v/v/v) and final pH adjust to 5.5±0.02 with phosphoric acid as the mobile phase. Calibration curves for both AMI and CLR were plotted and the corresponding regression equations were calculated.

For the HPTLC method, AMI and CLR stock solutions (500 μg/ml and 200 μg/ml, respectively) were prepared in methanol. These solutions (1.0 ml) were transferred into 2 separate 5 ml volumetric flasks and diluted to the mark with methanol to give final concentrations of 100 and 40 μg/ml, respectively. Different volumes of the working solutions (0.5, 1, 2, 3, 4, 5 and 6 μl, equivalent to 50, 100, 200, 300, 400, 500 and 600 ng/spot of AMI and 20, 40, 80, 120, 160, 200 and 240 ng/spot of CLR) were applied, in triplicate, to a TLC plate. Samples were applied to the plates as bands of width 5 mm, by means of a Camag (Switzerland) Linomat V sample applicator fitted with a Camag microlitre syringe. A constant application rate of 0.1 μl/s was used. Linear ascending development of the plates to a distance of 8 cm was performed with carbon tetrachloride:acetone:triethyl amine (6:3:0.2, v/v/v), as the mobile phase in a twin-trough glass chamber previously saturated with mobile phase vapour for 30 min at room temperature (25°). After development the plate was scanned at 240 nm by means of a Camag TLC scanner-3 in absorbance mode, using the deuterium lamp. The slit dimensions were 6×0.45 mm and the scanning speed was 20 mm/s. After development peak area data and drug concentration data were treated by linear regression to determine linearity.

Powder from the mixed contents of 20 tablets, equivalent to 25 mg AMI and 10 mg CLR, was transferred accurately to a 50 ml volumetric flask and diluted to volume with methanol. The solution was diluted to the same concentrations of working standard solutions and treated according to the linearity for the first derivative spectrophotometry, RP-HPLC and HPTLC methods.

The first derivative spectrophotometric method is used to eliminate the spectral interference from one of the two drugs while estimating the other drug by selecting the zero crossing point on the derivative spectra of each drug as the selected wavelength. Fig. 1 shows overlain first derivative spectra of AMI and CLR. AMI can be assayed in the presence of CLR by measuring absorption at zero crossing point of CLR in the range of 1-20 μg/ml. The linear regression Eqn was found to be: Y=0.0066X+0.0023, r= 0.9981, where Y is the absorbance value at 219 nm, X is the concentration in μg/ml and r is the correlation coefficient. CLR can be assayed in the presence of AMI by measuring absorption at zero crossing point of AMI in the range of 2-24 μg/ml. The linear regression Eqn was found to be: Y=0.0028X+0.003, r= 0.9994, where Y is the absorbance value at 230 nm, X is the concentration in μg/ml, and r is the correlation coefficient.

**Fig. 1:**
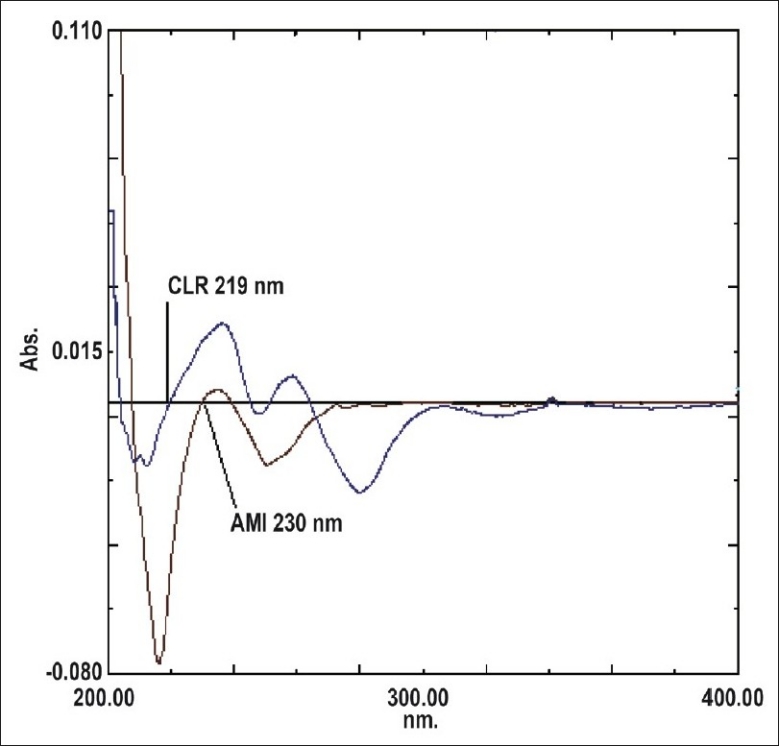
First derivative absorption spectra of AMI and CLR. AMI is amitriptyline HCl and CLR is chlordiazepoxide

To optimize the proposed RP-HPLC method, different systems were tried for chromatographic separation of the two components by combining homogenous design and solvent polarity optimization. The best resolution was achieved using a mobile phase consisting of methanol:acetonitrile:0.065 M ammonium acetate buffer (50:20:30, v/v/v) and final pH adjust to 5.5±0.02 with phosphoric acid, which even gave good sensitivity for both drugs (fig. 2). System suitability testing of the RP-HPLC method gave good relative retention time= 1.83; theoretical plates= 4640.66 and 6960; asymmetry factor (A)= 0.91 and 0.93; tailing factor (T)= 0.96 and 0.97 for AMI and CLR, respectively (Table 1). A linear relation was obtained between peak area and the concentration of the two drugs in the range of 0.25-4 and 0.1-1.6 μg/ml for AMI and CLR, respectively. The linear regression Eqns were, Y= 517021X−31721, r= 0.9999 and Y=1000000 X– 8895.6, r= 0.9997, where Y is the area under the peak, X is the concentration in μg/ml, and r is the correlation coefficient.

**Fig. 2:**
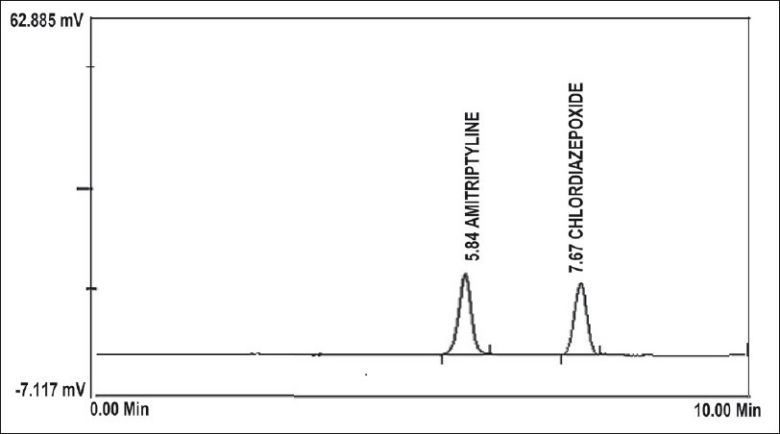
RP-HPLC chromatogram of AMI and CLR at 240 nm. AMI is amitriptyline HCl and CLR is chlordiazepoxide

**TABLE 1:** SYSTEM SUITABILITY TEST PARAMETERS FOR AMI AND CLR FOR PROPOSED RP-HPLC AND HPTLC METHODS

Parameters	Proposed methods			
	
	RP-HPLC		HPTLC	
		
	AMI± % RSD^a^	CLR± % RSD^a^	AMI± % RSD^a^	CLR± % RSD^a^
Retention time, min	5.84±0.25	7.67±0.15	-	-
Rf value	-	-	0.68±1.10	0.28±1.80
Tailing factor	0.96±0.86	0.97±0.92	-	-
Asymmetry factor	0.91±0.82	0.93±0.68	-	-
Theoretical plates	4641±0.64	6960±1.34	-	-
Repeatability of measurement (n^b^ = 6)	1.82	1.49	1.40	1.73

a RSD is the relative standard deviation

b n is number of determinations, AMI is amitriptyline HCl and CLR is chlordiazepoxide

To optimize the HPTLC parameters, several mobile phase compositions were tried. Carbon tetrachloride: acetone: triethyleamine (6:3:0.2, v/v/v), gave a sharp and symmetrical peaks of AMI and CLR with R_f_ values of 0.68±0.02 and 0.29±0.01, respectively (fig. 3). Well-defined spots (and peaks) were obtained when the chamber was saturated with mobile phase vapour for 30 min at room temperature (25°). A linear relation was obtained between peak area and the concentration of the two drugs in the range of 50-600 ng/spot and 20-240 ng/spot for AMI and CLR, respectively. The linear regression Eqns were, Y=8.0515X+37.333, r= 0.9996 and Y= 13.771X+88.616, r= 0.9952, where Y is the area under the peak, X is the concentration in μg/ml, and r is the correlation coefficient.

**Fig. 3:**
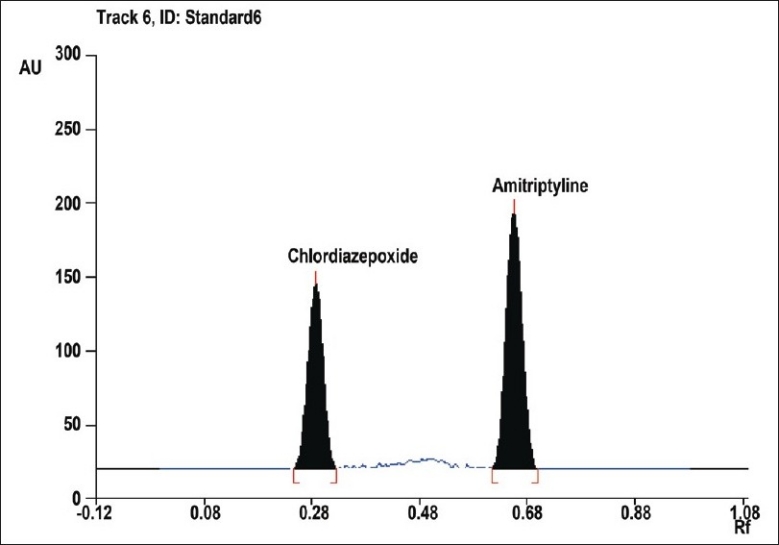
HPTLC Chromatogram of AMI and CLR at 240 nm. AMI is amitriptyline HCl and CLR is chlordiazepoxide

The proposed methods have been applied to assay AMI and CLR in tablets without any interference from the additives (Table 2). The validity of the suggested procedures was further assessed by applying the standard addition techniques (Table 3). The results of assay validation of the proposed methods show that they are accurate and precise according to the RSD values of intra and interday determinations (Table 4).

**TABLE 2:** ASSAY RESULTS FOR TABLETS USING THE PROPOSED METHODS

Formulation	Proposed methods	Mix.	Amount of drug added (mg)		Amount of drug found (mg)		% Amount found (n^a^=3) ± SD^b^	
					
			AMI	CLR	AMI	CLR	AMI	CLR
Tablets	First derivative UV	1	25	10	25.28	9.9	101.1±0.95	99±0.9
		2	25	10	25.38	9.8	101.5±1	98.4±0.95
	HPTLC	1	25	10	25.18	10.12	100.74±1.24	101.15±0.44
		2	25	10	25.12	10.09	100.47±0.79	100.92±0.35
	RP-HPLC	1	25	10	25.2	9.91	100.8±0.17	99.08±0.38
		2	25	10	25.19	9.94	100.76±0.61	99.41±0.47

a n is the number of determinations

b SD is the standard deviation, AMI is amitriptyline HCl and CLR is chlordiazepoxide

**TABLE 3:** APPLICATION OF THE STANDARD ADDITION TECHNIQUE TO THE ANALYSIS OF AMI AND CLR IN TABLETS BY THE PROPOSED METHODS

Proposed methods	Amount of drug taken		Amount of drug added		Amount of drug found		% Recovery (n^a^=3) ± SD^b^	
							
	(μg/ml or ng/spot)		(μg/ml or ng/spot)		(μg/ml or ng/spot)		
					
	AMI	CLR	AMI	CLR	AMI	CLR	AMI	CLR
First derivative UV	5	2	2.5	1	7.59	2.96	101.2±0.7	98.7±1.17
	5	2	5	2	10.13	3.97	101.3±1	99.3±1.04
	5	2	7.5	3	12.53	4.98	100.2±0.92	99.6±0.80
HPTLC	200	80	100	40	301.11	120.96	100.37±0.65	100.80±0.65
	200	80	200	80	400.65	160.94	100.16±1.03	100.58±1.45
	200	80	300	120	503.18	200.91	100.64±0.29	100.46±1.45
RP-HPLC	1	0.4	0.5	0.2	1.504	0.604	100.31±0.50	100.72±0.79
	1	0.4	1	0.4	2.014	0.806	100.7±1.16	100.79±0.58
	1	0.4	1.5	0.6	2.516	1.006	100.66±0.22	100.63±1.07

a n is the number of determinations

b SD is the standard deviation, AMI is amitriptyline HCl and CLR is chlordiazepoxide

**TABLE 4:** SUMMARY OF VALIDATION PARAMETERS FOR THE PROPOSED METHODS

Proposed methods	Drug	Parameters			
		
		LOD^a^	LOQ^b^	Interday (*n* = 3)	Intraday (n^d^ = 3)
		μg/ml	μg/ml	(RSD c, %)	(RSD c, %)
First derivative UV	AMI	0.33	1	1.49-3.26	1.10-2.86
	CLR	0.66	2	1.24-3.06	1.19-2.83
HPTLC	AMI	2.479916	7.51489	0.54-1.75	0.38-1.39
	CLR	0.44596	1.35139	0.62-1.67	0.33-1.44
RP-HPLC	AMI	0.0117	0.0357	1.07-1.83	0.65-1.22
	CLR	0.00227	0.0069	0.39-1.96	0.39-1.19

a LOD is the limit of detection

b LOQ is the limit of quantification

c RSD is the relative standard deviation

d n is the number of determinations, AMI is amitriptyline HCl and CLR is chlordiazepoxide

These methods were compared by applying the analysis of variance (ANOVA) test. The calculated F-value of 3.23 for AMI and 1.85 for CLR are less than the tabulated F-value (9.55) at the 95% confidence interval, which reveals that there is no significant difference with respect to accuracy and precision between the proposed methods. The proposed procedures can be applied for the simultaneous determination of AMI and CLR. Moreover, the methods are rapid, sensitive, accurate, precise and can be used in routine analysis.
